# Characterization of in vivo-acquired resistance to macrolides of *Mycoplasma gallisepticum *strains isolated from poultry

**DOI:** 10.1186/1297-9716-42-90

**Published:** 2011-08-02

**Authors:** Irena Gerchman, Sharon Levisohn, Inna Mikula, Lucía Manso-Silván, Inna Lysnyansky

**Affiliations:** 1 Mycoplasma Unit, Division of Avian and Fish Diseases, Kimron Veterinary Institute, Bet Dagan, 50250, Israel; 2 CIRAD, UMR CMAEE, F-34398 Montpellier, France

## Abstract

The macrolide class of antibiotics, including tylosin and tilmicosin, is widely used in the veterinary field for prophylaxis and treatment of mycoplasmosis. In vitro susceptibility testing of 50 strains of *M. gallisepticum *isolated in Israel during the period 1997-2010 revealed that acquired resistance to tylosin as well as to tilmicosin was present in 50% of them. Moreover, 72% (13/18) of the strains isolated from clinical samples since 2006 showed acquired resistance to enrofloxacin, tylosin and tilmicosin. Molecular typing of the field isolates, performed by gene-target sequencing (GTS), detected 13 molecular types (I-XIII). Type II was the predominant type prior to 2006 whereas type X, first detected in 2008, is currently prevalent. All ten type X strains were resistant to both fluoroquinolones and macrolides, suggesting selective pressure leading to clonal dissemination of resistance. However, this was not a unique event since resistant strains with other GTS molecular types were also found. Concurrently, the molecular basis for macrolide resistance in *M. gallisepticum *was identified. Our results revealed a clear-cut correlation between single point mutations A2058G or A2059G in domain V of the gene encoding 23S rRNA (*rrnA*, MGA_01) and acquired macrolide resistance in *M. gallisepticum*. Indeed, all isolates with MIC ≥ 0.63 μg/mL to tylosin and with MIC ≥ 1.25 μg/mL to tilmicosin possess one of these mutations, suggesting an essential role in decreased susceptibility of *M. gallisepticum *to 16-membered macrolides.

## Introduction

*Mycoplasma gallisepticum *is the major mycoplasma pathogen in poultry, causing Chronic Respiratory Disease in chickens and Infectious Sinusitis in turkeys [1]. In Israel, due to control measures, *M. gallisepticum *outbreaks occur infrequently in breeder flocks. However, *M. gallisepticum *is sometimes present in meat-type turkey flocks or other types of commercial flocks maintained for long growing periods with minimal biosecurity, which thereby may provide reservoirs of infection. Macrolides (tylosin, tilmicosin) and fluoroquinolones (enrofloxacin, difloxacin) are among the antibiotic families most widely used in poultry in many countries [2], including Israel.

Tylosin has been used prophylactically and therapeutically for mycoplasmosis in certain poultry sectors in Israel for more than 40 years [3]. Historically, tylosin was used for eradication of *M. gallisepticum *by egg-dipping at the same time as being used for control in the progeny, a practice that was linked to the emergence of *M. gallisepticum *tylosin-resistant strains, initially in turkey breeder flocks and later in broilers and meat-type turkeys [3]. With the successful eradication of *M. gallisepticum *in breeder flocks, there was a marked decrease in the use of tylosin in poultry and, probably as a consequence, only sporadic detection of *M. gallisepticum *tylosin-resistant strains [4]. Notably, tylosin-resistant *M. gallisepticum *strains, all with the same molecular profile identified by random amplification of polymorphic DNA analysis, were consistently isolated in one geographical region of Israel where tylosin-treated commercial flocks served as an environmental reservoir for outbreaks in breeder flocks [5].

The antibacterial activity of macrolides is due to inhibition of bacterial protein synthesis by binding to the 23S rRNA component of the bacterial 50S ribosomal subunit. Usually in bacteria with a small number of rRNA operons, such as mycoplasmas, acquired resistance to macrolides has been associated with mutations within domain II or V of the 23S rRNA genes or in *rplD *and *rplV*, genes encoding ribosomal proteins L4 and L22 [6].

Enrofloxacin has a fairly wide spectrum of efficacy and has been used as the routine choice for treatment of a variety of poultry diseases in addition to mycoplasmosis in many countries [2,7] including Israel where it was first introduced in the early 1990s. This practice may account for the relatively rapid emergence of enrofloxacin-resistance in clinical isolates of *M. gallisepticum *recently reported by our group [8] and others [9]. Molecular characterization of the quinolone resistance-determining regions (QRDRs) of DNA gyrase and topoisomerase IV in *M. gallisepticum *isolates with different levels of susceptibility to fluoroquinolones showed that enrofloxacin-resistant isolates harbor amino acid substitutions in the QRDRs of each of three proteins (GyrA, GyrB, and ParC) [10].

The present study reports on in vitro susceptibility to two macrolides (tylosin and tilmicosin) and to enrofloxacin in 50 *M. gallisepticum *clinical strains isolated in Israel during 1997-2010. In addition, by sequence analysis of domains II and V of the 23S rRNA genes as well as genes encoding ribosomal proteins L4 and L22 in clinical isolates with different levels of susceptibility to tylosin and tilmicosin, we investigated the mechanism of *M. gallisepticum *acquired-resistance to these two 16-membered macrolides. Furthermore, molecular typing by gene targeted sequencing (GTS) [11] was performed to characterize the *M. gallisepticum *strains isolated in Israel over time, attempting to gain insight into the emergence and dissemination of the resistance phenotype(s) in this population.

## Materials and methods

### *M. gallisepticum *strains and growth conditions

A total of 50 *M. gallisepticum *strains isolated during the period 1997-2010 from 15 meat-type turkey flocks (MT), 7 turkey breeder flocks (TB), 23 broiler breeder flocks (BB), 4 broiler flocks (B) and 1 Leghorn-type breeder flock (LB) were analyzed (Table 1). These include 24 isolates described previously [10]. In addition, reference strains *M. gallisepticum *PG31 (ATCC 19610), *M. gallisepticum *S6 (ATCC 15302), prototype pathogen *M. gallisepticum *R, and Israeli reference strain 227 were included in this study. Samples from breeder flocks (TB, BB and LB) were submitted to the laboratory within the framework of the national mycoplasma control program; clinical signs were not necessarily present. In contrast, samples from MT and B flocks were received by survey of flocks with respiratory disease and/or serological evidence of *M. gallisepticum *infection. The outbreaks had no known epidemiological link.

**Table 1:** In vitro sensitivity, GTS typing and molecular characterization of domain V in the 23S rRNA genes of *M. gallisepticum *clinical isolates.

No	Strain	**Sector **^**a**^	Year	Susceptibility			GTS type	**Domain V sequence**^**b**^	
						
				Ty	Tm	En		*rrnA*	*rrnB*
	S6			S	S	S	NA	A2058/A2059	A2058/A2059
	R			S	S	S	NA	A2058/A2059	A2058/A2059
	PG31			S	S	S	NA	NA	NA
	227	TB	1978	R	R	S	I	G2058/-	-/G2059
1	HZ-19	MT	1997	S	S	S*	II	-/-	-/G2059
2	NMH-2	TB	1997	R	R	S	III	-/G2059	-/G2059
3	BSY-10	B	1998	S	S	S*	IV	-/-	-/G2059
4	TY-6	MT	1998	R	R	S*	IV	-/G2059	-/G2059
5	DSD-4	MT	2000	R	R	S*	II	-/G2059	-/G2059
6	YS	BB	2001	S	S	S	II	-/-	-/G2059
7	EK	BB	2001	S	S	S	II	-/-	-/G2059
8	KBR-3	MT	2002	S	S	S*	II	-/-	-/G2059
9	KR-11	MT	2002	R	R	S*	II	-/G2059	-/G2059
10	TMM	BB	2002	S	S	S	II	-/-	-/G2059
11	KSH	BB	2002	S	S	S	II	-/-	-/G2059
12	SMR	TB	2002	S	S	S	V	-/-	-/-
13	BR	BB	2002	S	S	S	II	-/-	-/G2059
14	NLP	TB	2002	R	R	S	II	-/G2059	-/G2059
15	PYN	BB	2002	S	S	S	II	-/-	-/G2059
16	SBC	TB	2002	R	R	S	II	-/G2059	-/G2059
17	MKK	BB	2002	S	S	S	II	-/-	-/G2059
18	NR-3	LB	2003	R	R	S*	II	-/G2059	-/G2059
19	NH-7	TB	2003	R	R	S*	II	-/G2059	-/G2059
20	RFG	TB	2003	S	S	S	II	-/-	-/G2059
21	TR-9	MT	2004	R	R	S*	II	-/G2059	-/G2059
22	ABA-6	MT	2005	S	S	R*	II	-/-	-/G2059
23	BAK-2	BB	2005	S	S	S*	II	-/-	-/-
24	BNC-2	MT	2005	S	S	S*	VI	-/-	-/-
25	YDK-3	MT	2005	S	S	R*	II	-/-	-/G2059
26	MT-13	B	2005	S	S	R*	II	-/-	-/G2059
27	MYZ-8	BB	2005	S	S	R*	VII	-/-	-/G2059
28	SYR-3	BB	2005	S	S	R*	II	-/-	-/G2059
29	MKT-3	MT	2005	S	S	R*	II	-/-	-/G2059
30	SM-9	MT	2005	R	R	S*	II	-/G2059	-/G2059
31	MAR-1	B	2005	R	R	R*	II	-/G2059	-/G2059
32	KYN-6	BB	2005	R	R	S*	II	-/G2059	-/G2059
33	MSA-9	B	2006	S	S	R*	II	-/-	-/G2059
34	JL-10	MT	2006	S	S	R*	II	-/-	-/G2059
35	MDE-3	BB	2006	R	R	R*	VIII	-/G2059	-/G2059
36	RV-2	BB	2007	S	S	S*	IX	-/-	-/-
37	BLF-6	BB	2008	R	R	R	X	-/G2059	-/G2059
38	KFM	BB	2009	R	R	R	X	-/G2059	-/G2059
39	SU	MT	2009	R	R	R	XI	-/G2059	-/G2059
40	LH-22	MT	2009	R	R	R	X	-/G2059	-/G2059
41	VR-8	BB	2009	S	S	R	XII	-/-	-/-
42	KLD-8	BB	2009	R	R	R	X	-/G2059	-/G2059
43	CK-4	BB	2009	R	R	R	X	-/G2059	-/G2059
44	KC-4	BB	2009	R	R	R	X	-/G2059	-/G2059
45	KTY	BB	2009	R	R	R	VIII	-/G2059	-/G2059
46	TLS-2	BB	2010	S	S	S	XIII	-/-	-/-
47	MCK	BB	2010	R	R	R	X	-/G2059	-/-
48	MAT-394	BB	2010	R	R	R	X	-/G2059	-/G2059
49	MN-2	MT	2010	R	R	R	X	-/G2059	-/G2059
50	NLY	TB	2010	R	R	R	X	-/G2059	-/G2059

^a ^B = Broiler; BB = Broiler breeder; LB = Leghorn-type breeder; MT = Meat-type turkey; TB= turkey breeder.

^b ^Nucleotide present at positions 2058 and 2059.

"-", No polymorphism compared to the sequence of the reference strains.

* MIC values for En published in Gerchman et al. [13] and in Lysnyansky et al. [10].

NA = not analyzed.

All isolates were propagated at 37°C in Frey's broth medium. Isolates of *M. gallisepticum *were identified by direct immunofluorescence (IMF) of colonies using species-specific conjugated antiserum. Mixed cultures were cloned to IMF homogeneity by microscopic selection of target colonies. Notably, isolates were not filtered cloned in order to avoid inadvertent selection of a non-representative variant [12]. Aliquots of low-passage (3-4 p) cultures were stored at -80°C.

### Antimicrobial susceptibility testing

In vitro susceptibility for tylosin (98%, batch RS 0315), tilmicosin (91%, batch RS 0263), kindly provided by Eli Lilly (Indianapolis, IN, USA), and for enrofloxacin (98% active material, Fluka AG, Seelze, Germany) was determined by the microbroth dilution method as previously described [13], following the guidelines recommended by Hannan [14]. Two-fold dilutions of antibiotics from 0.04 - 10 μg/mL were tested. For *M. gallisepticum *strains sensitive to macrolides or to fluoroquinolones at the lowest concentration in the preliminary test, an additional round of testing with tylosin and tilmicosin or enrofloxacin in the range 0.0008 - 0.2 μg/mL was performed. The microbiological criterion (epidemiological cut-off value) was used for interpretation of MIC results [15].

MIC values for tylosin and tilmicosin in reference strains S6, R and PG31 were 0.0063 μg/mL and 0.0032 μg/mL, respectively for each of the strains (data not shown). The MIC values for enrofloxacin in reference strains S6, R and PG31 were 0.01, 0.005 and 0.01 μg/mL, respectively (data not shown). The MIC values for tylosin, tilmicosin, and enrofloxacin in this study were consistent with the previously published values for these strains [8,16-19].

The MIC values for Israeli reference strain 227 were 1.25 μg/mL for tylosin and tilmicosin and 0.05 μg/mL for enrofloxacin (data not shown).

### PCR amplification and sequence analysis of domains II and V of the 23S rRNA genes, *rplD *and *rplV *genes

*M. gallisepticum *genomic DNA was extracted from 10 mL logarithmic-phase broth cultures using the Maxwell^® ^16 apparatus and Maxwell 16™ Cell DNA Purification Kit (Promega, Madison, USA) according to the manufacturer's instructions. Primers used in this study were designed on the nucleotide sequence of *M. gallisepticum *strain R_low _(AE015450, [20]) (Table 2). Since the nucleotide sequences of the two *M. gallisepticum *23S rRNA genes (MGAr01 (*rrnA*) and MGAr04 (*rrnB*)) are highly homologous, four different PCR systems were designed to specifically amplify domains II and V in each gene. One of the primers in each PCR system (initial step) recognizes a sequence in the respective flanking gene (MGA_0754 to amplify domain V and MGA_0746 for domain II of MGAr01; MGA_1046 for domain V and MGAr03 for domain II of MGAr04) (Table 2). The primers MG23S-1F and MG23S-1R (for the nested reactions) contain a nucleotide sequence found in domain V of both MGAr01 and MGAr04 genes. Likewise, primers MG-rRNAII-F and MG-rRNAII-R (nested) recognize a nucleotide sequence in domain II of both MGAr01 and MGAr04 genes (Table 2). In addition, gene-specific primers corresponding to genes *rplD *and *rplV*, encoding ribosomal proteins L4 and L22, respectively, were synthesized (Table 2).

**Table 2:** Primers used for PCR amplification and sequencing of domains II and V of 23S rRNAs and ribosomal proteins of L4 and L22 in *M. gallisepticum*.

Primer designation	Source of primer	Sequence (5'-3')	**Nucleotide position**^**a**^	Amplicon size (bp)
**Domain V of MGAr01**				
MG-23S-1F^1^ MG-0754-R	MGAr01 MGA_0754	CACAGCTCTATGCTAAATCGC GATAATTGGTGGAGTTGG	82312-82332 and 324019-324039 83691-83708	1396
**Nested PCR for domain V of MGAr01**				
MG-23S-1F^1^ MG-23S-1R^2^	MGAr01 MGAr01	CACAGCTCTATGCTAAATCGC GGTCCTCTCGTACTAAG	82312-82332 and 324019-324039 83175-83191 and 324882-3244898	879
**Domain II of MGAr01**				
MG-mdh-F MG-rRNAII-R^3^	MGA_0746 MGAr01	GCAAGCACGGATGGAAGT CCACTGTCTGACTGCAAG	79928-79945 81483-81500 and 323190-323207	1572
**Nested PCR fordomain II of MGAr01**				
MG-rRNAII-F^4^ MG-rRNAII-R^3^	MGAr01 MGAr01	GGTTTAATACCTAGCAGGAT CCACTGTCTGACTGCAAG	80893-80912 and 322600-322619 81483-81500 and 323190-323207	607
**Domain V of MGAr04**				
MG-23S-1F^1^ MG-1046-R	MGAr04 MGA_1046	CACAGCTCTATGCTAAATCGC GCTAATTGCCTCCTGGTAAC	324019-324039 and 82312-82332 325669-325688	1669
**Nested PCR for domain V of MGAr04**				
MG-23S-1F^1^ MG-23S-1R^2^	MGAr04 MGAr04	CACAGCTCTATGCTAAATCGC GGTCCTCTCGTACTAAG	324019-324039 and 82312-82332 324882-324898 and 83175-83191	879
**Domain II of MGAr04**				
MG-16S-F MG-rRNAII-R^3^	MGAr03 MGAr04	GGAATCACTAGTAATCGC CCACTGTCTGACTGCAAG	321355-321317 323190-323207 and 81483-81500	1852
**Nested PCR for domain II of MGAr04**				
MG-rRNAII-F^4^ MG-rRNAII-R^3^	MGAr04 MGAr04	GGTTTAATACCTAGCAGGAT CCACTGTCTGACTGCAAG	322600-322619 and 80893-80912 323190-323207 and 81483-81500	607
***rplD *(L4)**				
MG-L4-F MG-L4-R	MGA_0710 MGA_0710	CGATTTATCTGGAAAAGTTCAAG GTTCAACCTTTCAACTCAGTTAT	67500-67522 68084-68106	606
***rplV *(L22)**				
MG-L22-F MG-L22-R	MGA_0716 MGA_0716	ATGATCGCAATTGCAAGACAA CTCCGCTAACTGATTGTTTTC	69631-69651 70034-70054	423

^a ^The primers' positions are based on the complete genome sequence of *M. gallisepticum *strain R_low _(AE015450) [20].

^1, 2, 3, 4 ^The primers contain the same nucleotide sequence.

PCR reaction mixtures contained 50 μL Ready-Mix PCR Master Mix (ABGene, Surrey, UK) with 30 pmol/μL of each primer (Sigma-Aldrich, Rehovot, Israel) and about 100 ng of mycoplasmal DNA. PCR amplifications were carried out in an MJ Research PT200 thermocycler (Waltham MA, USA). PCR conditions for primers MG-23S-1F/MG-0754-R and MG-23S-1F/MG-1046-R were the following: one cycle of 3 min at 95°C, 1 min 45 s at 56°C, 1 min 45 s at 72°C; 30 cycles of 95°C for 45 s, 56°C for 45 s, and 72°C for 1 min 45 s; 72°C for 10 min. To amplify domain II of the 23S rRNA genes (primers MG-mdh-F/MG-rRNAII-R or MG-16S-F/MG-rRNAII-R) the following program was used: one cycle of 3 min at 95°C, 2 min at 52°C, 2 min 30 s at 72°C; 30 cycles of 95°C for 45 s, 52°C for 45 s, and 72°C for 2 min; 72°C for 10 min. When the final PCR product of domain II or V of 23S rRNA was absent or was weak on the agarose gel, it was subjected to semi-nested PCR with primers MG-23S-1F/MG-23S-1R (for domain V) or with primers MG-rRNAII-F/MG-rRNAII-R (for domain II) as follows: one cycle of 3 min at 95°C, 45 s at 50°C, 1 min at 72°C; 30 cycles of 95°C for 30 s, 50°C for 30 s, and 72°C for 1 min; 72°C for 10 min. The nested program was also used for amplification of the *rplD *(primers MG-L4-F/MG-L4-R) and *rplV *(primers MG-L22-F/MG-L22-R) genes (Table 2). PCR products were visualized on ethidium bromide stained agarose gels, and then purified from the gel using the QIAquick Gel Extraction kit (QIAGEN, Hilden, Germany).

Sequencing was performed at the DNA Sequencing Unit, Weizmann Institute (Rehovot, Israel), utilizing the Applied Biosystems DNA Sequencer with the ABI BigDye Terminator Cycle Sequencing Kit (Applied Biosystems, Foster City, CA, USA). Sequence editing, consensus, and alignment construction were performed using DNASTAR software, version 5.06/5.51, 2003 (Lasergene, Inc. Madison, Wisconsin, USA).

Numbering of the nucleotide substitutions in domain V of the 23S rRNA sequenced amplicon is according to the sequence of the respective genes in *Escherichia coli*.

### GTS typing

Molecular typing was performed by modified GTS analysis [11]. The *pvpA, gapA*, and *lp *(MGA_0319) partial gene sequences were amplified using primers pvpA 4F/3R, gapA 3F/4R, and lp 1F/1R described previously [11]. However, the *mgc2 *gene was amplified using primers *mgc2 *2F/2R, previously described by Garcia et al., [21], resulting in an amplicon of about 300 bp. Amplified gene fragments of the respective genes were sequenced as described above.

The sequences obtained from each corresponding forward and reverse primer were assembled using the SeqMan program (Lasergene, DNASTAR) and the extremities showing single strand sequences, as well as aberrant sequences, were trimmed. All sequences obtained for each gene were aligned using Clustal V (Megalign, Lasergene, DNASTAR, Madison, Wisconsin, USA) and trimmed to the same size for diversity analysis. Phylogenetic trees for individual genes were constructed from the Clustal V alignments by the neighbor-joining method and 1000 bootstrap replicate analysis using the MEGA 5 software [22,23]. In contrast with the previously published GTS analysis, in which *mgc2 *and *pvpA *gene fragments were 584 bp and 455 bp-long, respectively [11], *mgc2 *and *pvpA *sequences were 300 bp and 700 bp-long, respectively (sizes corresponding to the *M. gallisepticum *Rlow genome [20]). The different sequences obtained for each gene fragment were assigned different allele numbers, designated by Arabic numerals (1, 2, 3, etc; data not shown). The GTS types, based on the allelic profiles of the four genes, were designated by Roman numerals (I, II, III, etc) (Table 1).

The four gene sequences corresponding to each of the GTS types identified amongst the 51 strains analysed, were concatenated head-to-tail for diversity analysis using Darwin 5.0 [24] available at [25]. A distance tree was constructed using the neighbor-joining algorithm with the "simple matching" option (no correction applied to dissimilarities). The "pairwise gap block correction" option was selected with a minimal length for gap blocks of one nucleotide. This implied that all consecutive gaps, starting from one nucleotide, were considered as a single event. A bootstrap analysis with 1000 replicates was performed to test the stability on randomly chosen sets of positions.

Sequences of 50 *M. gallisepticum *isolates and 1 Israeli reference strain were submitted to GenBank under the following accession numbers: *gapA*, JN102573-102623; MGA_0319, JN102624-102674; *pvpA*, JN113291-113341; *mgc2*, JN 13342-113392.

## Results

### Antimicrobial susceptibility

An overview of the MIC values, MIC_50_, and MIC_90 _for tylosin, tilmicosin, and enrofloxacin for the 50 strains tested is presented in Table 3. Bimodal distribution of MIC was identified for all three antimicrobial agents, indicating acquired resistance in isolates in the higher range of MIC values.

**Table 3:** Distribution of minimal inhibitory concentration (MIC) for tylosin, tilmicosin, and enrofloxacin among 50 *M.gallisepticum *field strains, determined by the microbroth dilution method.

Number of isolates with MIC (μg/mL) of															
**Antimicrobial agent**	≤**0.0032**	**0.0063**	**0.0125**	**0.025**	**0.05**	**0.1**	**0.25**	**0.63**	**1.25**	**2.5**	**5**	≥**10**	**MIC**_**50**_	**MIC**_**90**_	**% Resistance**

Tylosin	1	11	10	2	1			**3**	**8**	**13**	**1**		0.05	2.5	50
Tilmicosin	16	6		2		1			**3**		**7**	**15**	0.1	≥10	50
Enrofloxacin*				3	10	9	5		**2**	**11**	**8**	**2**	0.25	5	46

*M. gallisepticum *strains considered to have acquired resistance according to the microbiological criterion are represented in bold.

* MIC values for enrofloxacin are presented for 26 *M. gallisepticum *strains tested in this study and for 24 strains tested by the same method with the same reference strains and controls and published previously [6,8].

In Table 1 susceptibility data are presented on an individual strain basis indicating year of isolation and poultry sector of origin.

### The molecular basis of macrolide resistance in *M. gallisepticum *clinical isolates

No nucleotide mutations associated with decreased susceptibility to macrolides were identified in the *rlpD *and *rlpV *genes or in domain II of the 23S rRNA genes (data not shown). However, sequence analysis of domain V revealed that all 25 *M. gallisepticum *tylosin and tilmicosin-resistant clinical isolates had nucleotide transitions in one or both 23S rRNA genes (Table 1). Twenty-four tylosin and tilmicosin-resistant *M. gallisepticum *strains showed an A2059G transition in both *rrnA *and *rrnB *and strain MCK showed an *rrnA *A2059G transition (Table 1). The Israeli reference strain 227 with MIC of 1.25 μg/mL to tylosin and to tilmicosin had *rrnA *A2058G and *rrnB *A2059G transitions. In the cohort of 25 tylosin- and tilmicosin-susceptible clinical isolates, 19 possessed only the *rrnB *A2059G substitution and 6 did not show any substitution (Table 1). Notably, no increase in the MIC values was found between cohorts of *M. gallisepticum *isolates showing the genotype found in reference strains S6 and R (*rrnA *A2058A/A2059A and *rrnB *A2058A/A2059A) and *M. gallisepticum *strains possessing the single nucleotide substitution A2059G in *rrnB *(Table 1).

### Molecular typing of *M. gallisepticum *clinical isolates

**Figure 1:**
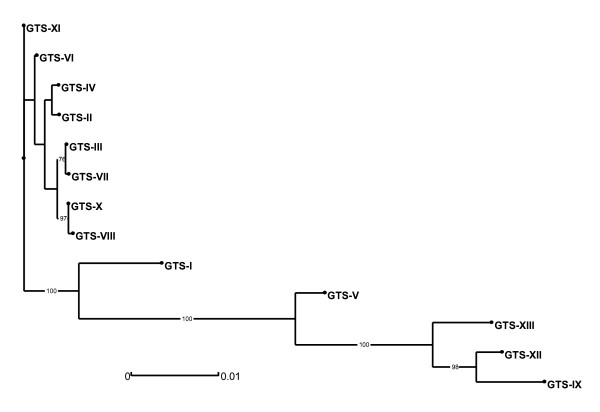
**Phylogenetic tree derived from distance analysis of the four concatenated GTS partial gene sequences**. The tree was constructed using the neighbor-joining algorithm (Darwin 5.0). A single sequence representing each of the 13 GTS types (I to XIII, Table 1) is displayed. Bootstrap percentage values were calculated from 1000 resamplings and values over 70% are displayed. The scale bar shows the distance equivalent to 1 substitution per 1000 nucleotide positions.

The genetic variability of Israeli *M. gallisepticum *strains was assessed by a modification of the previously described GTS method [11]. Overall, 13 GTS groups, arbitrarily designated I through XIII, were distinguished by analysis of the partial gene sequences of *mgc2*, *pvpA*, *gapA*, and *lp *(Table 1 and Figure 1). The results show that 38 of the 51 strains analyzed belong to two major groups: GTS type II, comprising 28 isolates, and GTS type X, with ten isolates. Type II was identified in 28/35(80%) of *M. gallisepticum *strains isolated between 1997 and 2006 and was not identified after 2006 (Table 1, No. 1-35). Type X, first detected in 2008, was found in 10/14 (71%) of the strains isolated since then (Table 1, No. 37-50). Type X differs from type II by the nucleotide sequences of three genes (*mgc2*, *pvpA*, and *lp*). Two isolates each possess GTS type IV and GTS type VIII. The remaining nine strains were found to have unique patterns. Interestingly, GTS type X is very closely related to type VIII (Figure 1). They diverge only by a single nucleotide polymorphism (SNP) in the *pvpA *sequence, namely by 1 out of 1837 nt of the concatenated sequences analyzed (0.054% divergence). GTS type VIII was first identified in 2006 and was found again in 2009.

Notably, the susceptibility profiles of *M. gallisepticum *strains presented in Table 1 show that isolates with molecular type II vary with respect to susceptibility to tylosin and enrofloxacin (11 strains susceptible to both antibiotics, nine resistant to tylosin and susceptible to enrofloxacin, seven susceptible to tylosin and resistant to enrofloxacin, and one resistant to both antibiotics). In contrast, all the *M. gallisepticum *strains with molecular types VIII and X were resistant to enrofloxacin as well as to tylosin and to tilmicosin (Table 1).

## Discussion

In the present study, the microbiological criterion was used for interpretation of MIC results, since no Clinical and Laboratory Standards Institute breakpoints for tylosin, tilmicosin and enrofloxacin are available for the avian pathogen *M. gallisepticum *[26]. The bimodal distribution of the cohort of strains tested (Table 3) allows wild-type susceptible populations of bacteria to be distinguished from those with acquired resistance [15,27]. Indeed, according to the data presented in Table 3, *M. gallisepticum *field strains with MIC of ≥ 0.63 μg/mL to tylosin, ≥ 1.25 μg/mL to tilmicosin and ≥ 1.25 μg/mL to enrofloxacin should be considered as resistant strains. The difference between susceptible and resistant populations for macrolides is more than tenfold (0.05 vs. ≥ 0.63). Using this criterion, 50% of all *M. gallisepticum *strains checked in this study were resistant to tylosin and tilmicosin and 46% were resistant to enrofloxacin (Table 3). Moreover, 12/14 (86%) and 13/14 (93%) of recently isolated *M. gallisepticum *strains (2008-2010) were resistant to tylosin and tilmicosin and to enrofloxacin, respectively (Table 1). Resistance to tylosin is not a new phenomena in Israel, being present at least sporadically since the 1970s [3]. Moreover, tylosin-resistant *M. gallisepticum *strains have been previously isolated under field conditions in many countries [9,19,28-30]. In contrast, although enrofloxacin has been used in local poultry since the early 1990s, enrofloxacin-resistant *M. gallisepticum *strains were first detected in 2005. However since then, 23/29 (79%) of the *M. gallisepticum *field strains tested were resistant to enrofloxacin (Table 1). The emergence of widespread resistance to tylosin and tilmicosin in clinical isolates of *M. gallisepticum *in Israel, often together with resistance to enrofloxacin, may confound treatment of mycoplasma infection in poultry.

The data presented herein demonstrate that a single point mutation in domain V of the 23S rRNA gene of operon *rrnA *is highly correlated with decreased susceptibility of *M. gallisepticum *to 16-membered macrolides (Table 1). Indeed, with one exception, all isolates with a MIC of ≥ 0.63 μg/mL to tylosin and a MIC of ≥ 1.25 μg/mL to tilmicosin show the transition A2059G in the *rrnA*. Interestingly, the single exception is *M. gallisepticum *strain 227 that has the nucleotide transition A2058G in domain V of 23S rRNA gene of *rrnA *(Table 1). This strain was isolated more than 30 years ago and possesses a unique GTS-pattern. No correlation between the presence of nucleotide substitution A2059G in *rrnB *and acquired resistance or decreased susceptibility to tylosin was found in this study.

It has been shown previously that tylosin-resistant *M. gallisepticum *mutants selected in vitro by erythromycin harbored an A2058G substitution in one of the two 23S rRNA genes [31]. However, since the authors did not use the designation *rrnA *and *rrnB*, it was not possible to clarify in which 23S rRNA gene the A2058G mutation was identified. In addition, among the tylosin-resistant mutants selected by erythromycin, a G2057A mutation and an A2059G mutation were found in one of the 23S rRNA genes. In the same study, in *M. gallisepticum *tilmicosin-selected tylosin-resistant mutants, two mutations, A2058G and A2503U, occurred in one of the two 23S rRNA genes and those mutants were characterized by markedly high resistance [31]. In another study, in vitro selection of *M. gallisepticum *mutants resistant to tiamulin, a member of the pleuromutilin family of antibiotics that also bind at the peptidyl transfer site in the 23S rRNA, resulted in nucleotide substitutions within domain V of the 23S rRNA genes [32]. Mutants with the A2058G or the A2059G mutation showed cross-resistance to erythromycin, tilmicosin and tylosin. Interestingly, 1/3 of these mutants harbored A2058G substitution in the *rrnB *gene, while the other two have A2058G or A2059G in the *rrnA *gene [32]. The nt substitutions at 2058 or at 2059 have been previously reported as hot spots for macrolide resistance in other bacteria and mycoplasmas [6,33-39].

Molecular typing of the 50 Israeli isolates analyzed in this study revealed the presence of two predominant types: GTS type II dominated until 2006 and type X has been dominant since first detected in 2008. By molecular analysis of the nucleotide sequences of *pvpA*, *lp*, and *mgc2 *genes, type X is relatively distant from type II but very closely related to VIII and may be considered as part of the same clonal complex.

Interestingly, all *M. gallisepticum *strains of the currently dominant GTS type X (as well as type VIII) are resistant to tylosin, tilmicosin, and enrofloxacin, suggesting clonal dissemination of this phenotype (Table 1). However, this apparently was not a unique occurrence, as evidenced by the presence of a resistant strain with a different GTS pattern (Table 1, No.39).

In conclusion, our study shows the recent emergence of acquired resistance to both the macrolide and fluoroquinolone classes of antibiotics in *M. gallisepticum*, mainly present in field isolates closely related by molecular typing. A comparison between cohorts of *M. gallisepticum *tylosin- and tilmicosin-resistant and -susceptible field strains revealed that acquired resistance to tylosin and tilmicosin may be attributed to mutations A2058G or A2059G in domain V of 23S rRNA gene (operon *rrnA*). This is the first report of a mechanism for macrolide-resistance in *M. gallisepticum *clinical isolates. A comparison of MIC in organisms with genetically characterized resistance mutation/s may offer a feasible way to validate MIC breakpoint values. Therefore, the data presented in this paper may help establish a workable breakpoint for macrolides in *M. gallisepticum*. Characterization of domain V of the 23S rRNA genes in a greater number of *M. gallisepticum *macrolide-resistant clinical isolates, especially those originating from other countries, will help elucidate whether the nt mutations observed in this study are characteristic only of this cohort of strains or are the universal markers for macrolide resistance in this pathogenic mycoplasma.

## Competing interests

The authors declare that they have no competing interests.

## Authors' contributions

IG carried out MIC assays, molecular analyses and participated in sequence processing. SL participated in the design of the study, manuscript preparation and its editing. IM participated in molecular analyses and PCR testing. LM-S performed diversity analyses and construction of phylogenetic trees and participated in manuscript preparation. IL designed and coordinated the study and drafted the manuscript. All authors read and approved the final version of the manuscript.
